# Anxiolytic-Like and Sedative Effects of *Alcea Aucheri* (Boiss.) Alef. Flower Extract in the Laboratory Rat

**Published:** 2017

**Authors:** Tajmah Mombeini, Hamid Gholami Pourbadie, Mohammad Kamalinejad, Soroush Mazloumi, Ahmad Reza Dehpour

**Affiliations:** a *Department of Pharmacology, School of Medicine, Shahed University, Tehran, Iran. *; b *Neuroscience Research Center, Shahid Beheshti University of Medical Sciences, Tehran, Iran. *; c *Department of Physiology and Pharmacology, Pasteur Institute of Iran, Tehran, Iran. *; d *School of Pharmacy, Shahid Beheshti University of Medical Sciences, Tehran, Iran. *; e *School of Medicine, Shahed University, Tehran, Iran.*; f *Experimental Medicine Research Center, Tehran University of Medical Sciences, Tehran, Iran. *; g *Department of Pharmacology, School of Medicine, Tehran University of Medical Sciences, Tehran, Iran.*

**Keywords:** Anxiolytic-like effect, *Alcea aucheri* (Boiss.) Alef., Elevated plus-maze, Open field, Total phenols, Total flavonoids

## Abstract

The present study was conducted to investigate the possible anxiolytic and sedative of an acute administration and 4-day repeated dosing of an aqueous *extract of flowers of Alcea aucheri* (Boiss.) Alef. (EFA)in rats subjected to the elevated plus-maze (EPM), open-field, and horizontal wire tests. All drugs were administered intraperitoneally. Phytochemical screening confirmed the presence of phenolic compounds, flavonoids, and polysaccharides in the extract. Repeated dosing of EFA (at dose of 35 mg/kg) significantly increased percentage of time spent on open arms and of open arms entries, and also decreased percentage of time spent on closed arms and of closed arms entries; compared with saline control, 24 h after treatment. In addition, repeated dosing of EFA (at dose of 175 mg/kg) significantly increased open arm activity 48 h after treatment, versus saline group. This effect was also observed following acute administration of EFA at 175 mg/kg*.* In open field, acute administration of EFA at doses of 17.5, 35, 70, 175, 350, and 700 mg/kg induced a statistically significant and dose-dependent decrease in locomotor activity, compared with saline control. ED50 value for EFA-induced decrease in locomotor activity was 194 mg/kg. Furthermore, unlike diazepam; EFA didn´t decrease the percent of the rats grasping the wire.

These data suggest that *Alcea aucheri* extract may have anxiolytic and sedative properties and some of the components in the extract such as phenolic compounds may have contributed to the observed effects.

## Introduction

Anxiety disorders are among the most common psychiatric disorders that affect all age groups of the general population (1). These disorders are widely treated with benzodiazepine anxiolytic agents. However, the clinical use of benzodiazepines is limited by their side effects such as respiratory depression, motor coordination deficits, memory/cognitive dysfunctions, and dependence liability (2-4). Therefore, finding novel therapeutic agents with fewer complications in the treatment of anxiety disorder, is of major interest to researchers (5). Medicinal herbs with traditional background of use in neurological diseases could be good candidates to find new anxiolytic agents.

Plants of the *Alcea* (or* Althaea*) genus from *Malvaceae* family are among important medicinal plants in Iranian traditional medicine. They have long been used in the treatment of health problems and diseases. The flowers of *Alcea spp*. have been widely used as mucilage for treatment of irritated oral and pharyngeal mucosa, respiratory, and gastrointestinal disorders, as well as urinary complaints and skin inflammations. They have been also used as a diuretic agent and sedative remedy (6, 7). Some of species of the *Alcea* genus with medicinal activities include* Alcea rosea L. *(hollyhock)*,* *A. officinalis L. *(marshmallow), and* A. aucheri *(Boiss.) Alef. (South marshmallow) (6). Literature survey revealed a variety of pharmacological actions such as antioxidant, antibacterial, antineoplastic, cough suppressive, immuno-stimulatory, and modulatory effects on hormonal activity for these plants; supporting traditional knowledge (8-13). As it was mentioned, these plants have been used as a sedative remedy in alternative medicine (6). However, this property remains to be investigated; neither clinical nor experimental assessments are present to indicate the sedative or anxiolytic effect for the *Alcea* spp. 

Therefore, the present study was undertaken to assess the possible anxiolytic and sedative effects following repeated or single administration of aqueous extract of flower of *A. Aucheri* (EFA) in rats. For this purpose, we used the elevated plus-maze and open field tests. Additionally, the effect of the *Alcea* extract on muscle tone was evaluated using the horizontal wire test.

## Experimental

*Chemicals and reagents*

All chemicals used were of analytical grade. Quercetin dehydrate, gallic acid, anhydrous sodium carbonate (Na2CO3), aluminum tri chloride, potassium acetate, sodium acetate, Folin–Ciocalteu reagent, mercuric chloride, potassium iodide, and iodine were purchased from Sigma–Aldrich (St. Louis, MO). Ethanol, methanol, hydrochloric acid (HCl), sulfuric acid (H2SO4), chloroform, ammonia, glacial acetic acid, and sodium hydroxide (NaOH) were purchased from Merck and potassium peroxodisulfate from Fluka. All chemicals and reagents were used without further purification.

**Table 1:** Total content of polysaccharides, flavonoids and phenolic compounds in the aqueous extract of *Alcea aucheri (*EFA)

**Phenolic compounds** c (mg of GAE/g )	**Flavonoids** b (mg QE/g)	**Polysaccharides** a (%) (w/w)	
136.3 ± 4.4	0.003 ± 0.0002	0.4	Aqueous extract

Data are expressed as mean (polysaccharides) or mean ± SD (flavonoids and phenolic compounds) (n = 3).

a The data is the percentage of total polysaccharide based on glucose calibration curve in gram per 100 gram extract (w/w).

b The data is expressed as quercetin equivalents (QE) in milligrams per gram extract.

c The data is expressed as gallic acid equivalents (GAE) in milligrams per gram extract.

**Table 2:** Effects of the aqueous extract of *A. aucheri* flowers (EFA) on locomotor measures in the elevated plus-maze (single dose, repeated doses

**Locomotor measures**	**Saline**	**Aqueous extract of A. ** ***aucheri*** **: EFA (mg/kg)**			**Diazepam (mg/kg)**	***P***
		**175**	**70**	**35**	**1.2**	
**No. of closed entries**						
Single-1 h Repeated-24 h Repeated-48 h Repeated-96 h	8.18 ± 1.21 17.67 ± 2.70 14.67 ± 2.38 13.25 ± 1.93	10.47 ± 1.36 13.67 ± 1.72 10.33 ± 1.44 12.40 ± 2.90	11.00 ± 1.03 13.33 ± 3.20 12.75 ± 1.62 10.75 ± 1.79	7.72 ± 1.49 10.17 ± 1.64 12.17 ± 1.39 13.75 ± 2.68	NS NS NS NS	9.11 ± 1.26 15.83 ± 2.24 11.25 ± 1.43 12.80± 2.24
**Total arm entries**						
Single-1 h Repeated-24 h Repeated-48 h Repeated-96 h	29.00 ± 3.82 49.00 ± 7.43 43.25 ± 5.75 42.00 ± 3.31	35.73 ± 3.51 42.50 ± 5.26 32.25 ± 3.98 37.8 0 ± 6.71	33.61± 2.67 41.83 ± 6.56 38.25 ± 3.65 30.60 ± 3.60	25.83 ± 3.79 42.67 ± 4.04 35.17 ± 3.88 43.50 ± 8.21	NS NS NS NS	31.00± 3.31 42.00 ± 3.67 40.00 ± 6.27 37.8 ± 3.26
**Distance (cm)**						
Single-1 h Repeated-24 h Repeated-48 h Repeated-96 h	‎1301‎ ±‎ 87.19‎ 1658 ± 64.2 1792 ± 119 1876 ± 27.29	‎1379 ‎±‎98.74‎ 1295 ± 71.39 **††‎‎ 1649 ± 84.17 1479 ± 113	‎1492 ‎±‎ 66.77‎ 1471 ± 19.93 1765 ± 62.85 1656 ± 83.09	‎1313 ± 75.83‎ 1602 ± 46.48 1691 ± 53.94 1659 ± 72.17	NS 0.002 NS NS	‎1587 ‎± ‎61.41‎ 1417 ± 77.12* 1667 ± 85.65 1819 ± 181.1
**Velocity (cm/sec)**						
Single-1 h Repeated-24 h Repeated-48 h Repeated-96 h	5.52 ± 0.24 6.02 ± 0.37 6.27 ± 0.37 6.27 ± 0.09	5.57 ± 0.22 4.76 ± 0.31** 5.75 ± 0.24 5.21 ± 0.39	5.70 ± 0.23 5.10 ± 0.12 5.95 ± 0.21 5.57 ± 0.27	5.52 ± 0.40 5.76 ± 0.13 5.70 ± 0.17 5.55 ± 0.25	NS 0.005 NS NS	5.47± 0.31 5.74 ± 0.12 5.83 ± 0.29 6.21 ± 0.72

Locomotor activity made by animals in the elevated plus-maze was automatically recorded by Ethovision software. Data are expressed as mean ± SEM of 10-12 rats.

** *p *< 0.01*. *

* *p *< 0.05 compared with saline control group*; *

† † *p *< 0.01 compared with EFA at dose of 35 mg/kg.

**Figure 1:**
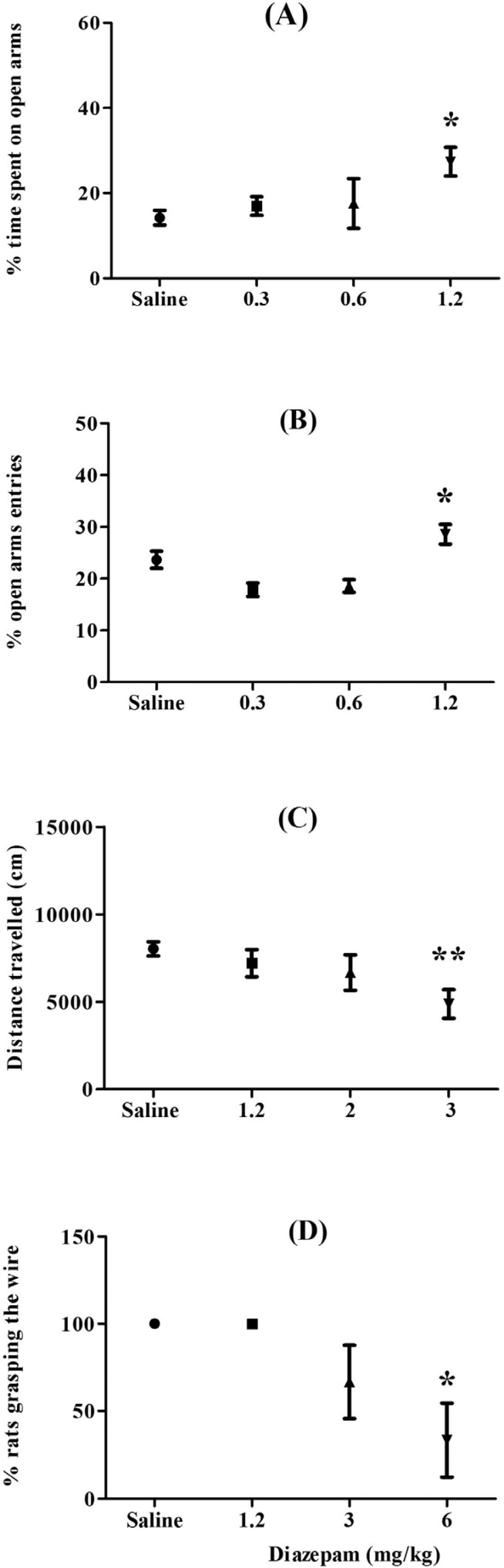
Effects of different concentrations of diazepam (IP) on the elevated plus-maze (A and B), open field (C), and horizontal wire test (D). In the EPM rats were tested 1 h following injection, in open field immediately after injection, and in horizontal wire test, immediately after open field. Bars represent the mean ± SEM, with n = 6, *p < 0.05, **p < 0.01 compared with saline group

**Figure 2:**
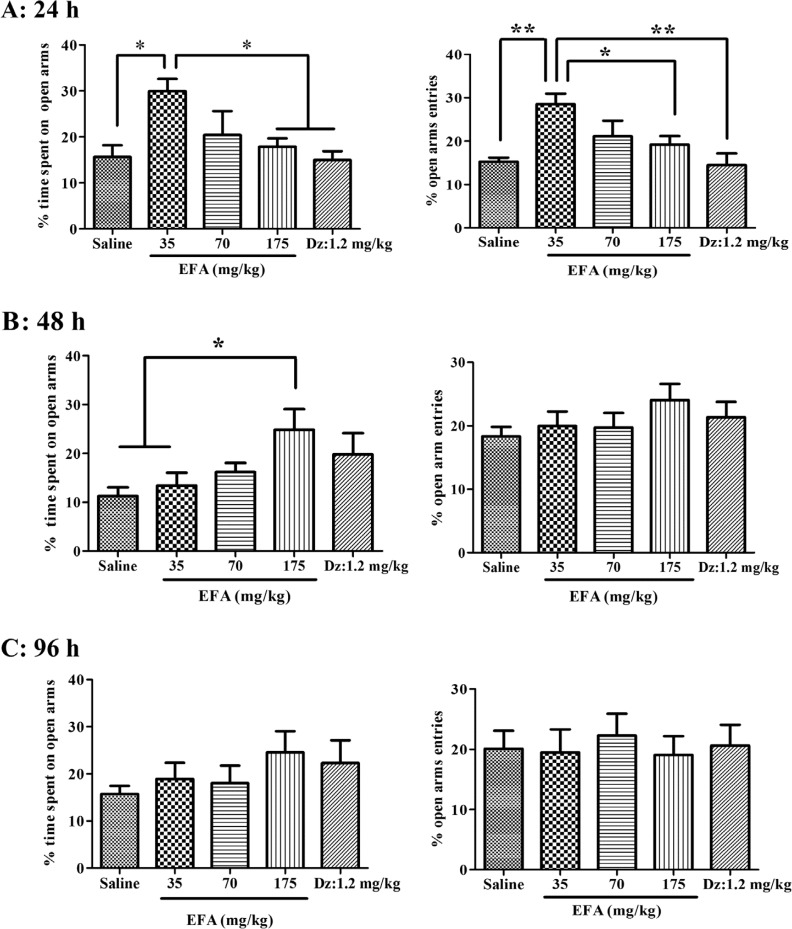
Effects of repeated dose administration of the aqueous extract of *Alcea aucheri* (EFA) on the elevated plus-maze test (EPM). Drugs were administered intraperitoneally to rats, once daily, for 4 days. Then rats were subjected to the elevated plus-maze with interval of 24 h, 48 h, or 96 h after the last dose [in Repeated-24 h (panel A), Repeated 48 h (panel B) or repeated 96 h (panel C) group; respectively]. The percentage of time spent on open arms and percentage of open arm entries were measured during a 5 min period. Each bar indicated the mean ± SEM of 10-12 treatment rats. Dz: diazepam; *p < 0.05, **p < 0.01.

**Figure 3:**
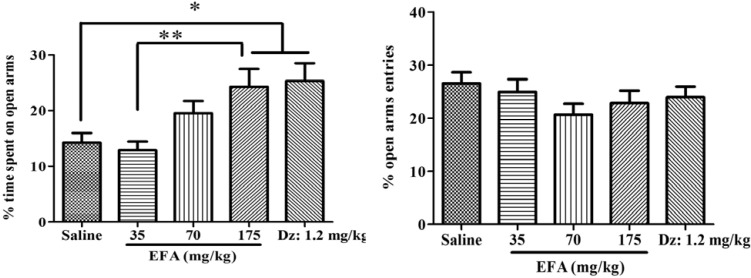
Effects of single dose administration of the aqueous extract of *Alcea aucheri* (EFA) on the elevated plus-maze test (EPM). Rats were subjected to the elevated plus-maze an hour after injection. Then percentage of time spent on and entries into open arms were measured during a 5 min period. Each bar indicated the mean ± SEM of 12 treatment rats. Dz: diazepam; *p < 0.05, **p < 0.01

**Figure 4:**
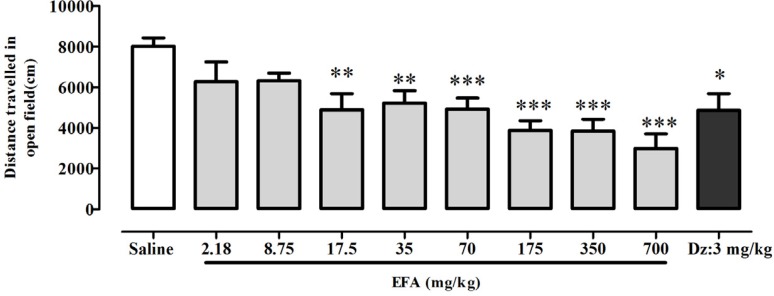
Effects of aqueous extract of *Alcea aucheri* (EFA) on spontaneous locomotor activity. Immediately after injection of diazepam or extract of* A. aucheri*, locomotor activity was measured as distance (cm) travelled by rat during 30 min, in the open field test. Each bar indicated the mean ± SEM of 7 treatment rats. Dz: diazepam; *p < 0.05, **p < 0.01, ***p < 0.001 compared with saline group

**Figure 5:**
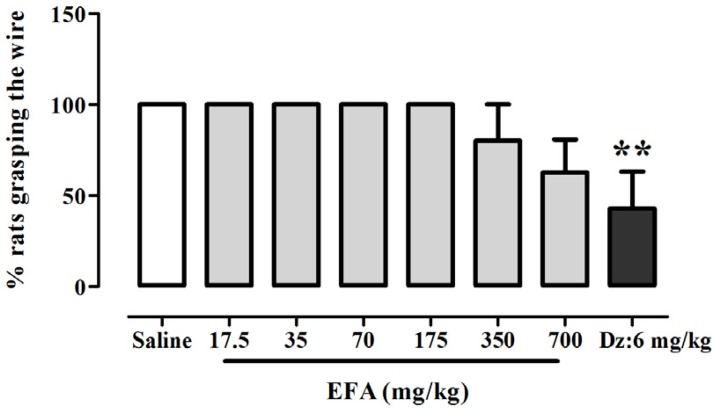
Effects of aqueous extract of *Alcea aucheri* (EFA) on the muscle tone of rats in the horizontal wire test. Each bar indicated the mean ± SEM of 7 treatment rats. Dz: diazepam; *p < 0.01 compared with control group

*Plant materials*

The fresh whole herb of *Alcea aucheri *was collected from Meydavoud village (Khuzestan province, Iran) on March 2015. The identity of the herb was confirmed by Department of Pharmacognosy, School of Pharmacy, Shahid Beheshti University of Medical Sciences, Tehran, Iran. A voucher specimen (SBMU-8021) was kept in the herbarium of School of Pharmacy for future reference.

*Preparation of aqueous extract*

The fresh and healthy flowers were separated instantly, and then were washed with natural water twice and dried under shade at room temperature for 3 d. The dried flowers (100 g) were ground using a grinder for 30 sec. Then, the powdered *A. aucheri *was macerated using 1000 mL of boiling distilled water, and allowed to infuse for 2 h at room temperature. The extract was filtered, then concentrated over the water bath, brought to dryness under vacuum, and stored at 4 °C in refrigerator until use. The extract yield was 7.2% (w/w).

*Phytochemical screening*

Phytochemical screening was carried out to identify alkaloids, flavonoids, saponins, tannins, phenolic acids, sterols, cardiac glycosides, and carbohydrates present in the aqueous extract of flower of *A. aucheri* (14-16‎).

*Alkaloids*

The aqueous extract was evaporated to dryness and the residue was heated on a boiling water bath with 2N HCl (5 mL). After cooling, the mixture was filtered and the filtrate was divided into two equal portions. One portion was treated with a few drops of Mayer´s reagent and the other with equal amounts of Wagner´s reagent. The samples were then observed for the presence of turbidity or precipitation. A (+) score was recorded if the reagent produced only a slight opaqueness; a (++) score was recorded if a definite turbidity, but no flocculation was observed and a (+++) score was recorded if a definite heavy precipitate or flocculation was produced.

*Flavonoids*

The aqueous extract was treated with a few drops of concentrated HCl and magnesium turnings (0.5 g). The presence of flavonoids was indicative if the pink or magenta-red color developed within 3 min.

*Saponins*

The aqueous extract was dissolved in boiling water. After cooling, the extract was shaken vigorously to froth and was then allowed to stand for 15-20 min and classified for saponin content as follows: no froth = negative; froth less than 1 cm = weakly positive; froth 1.2 cm high = positive; and froth greater than 2 cm high = strongly positive.

*Tannins and phenolic compounds*

The aqueous extract was evaporated and the residue was extracted by 10 mL of hot 0.9% NaCl solution, filtered, and divided into 3 equal portions. A sodium chloride solution was added to one portion of the text extract, 1% gelatin solution to a second portion and the gelatin-salt reagent to a third portion. Precipitation with the latter reagent or with both the second and third reagent is indicative of the presence of tannins. Positive tests are confirmed by the addition of FeCl3 solution to the extract and should result in a characteristic blue, blue-black, green or blue green color and precipitate (phenolic compounds, *i.e.* phenolics).

*Steroids*

The aqueous extract (100 mg) was shaken with chloroform in a test tube; few drops of acetic anhydride was added to the test tube and boiled in a water bath and rapidly cooled in iced water. Concentrated H2SO4 (2 mL) was added alongside of the test tube. Formation of a brown ring at the junction of two layers and turning the upper layer to green shows the presence of steroids.

*Cardiac glycosides*

The aqueous extract (0.5 g) was shaken with distilled water (5 mL). To this, glacial acetic acid (2 mL) containing a few drops of ferric chloride was added, followed by H2SO4 (1 mL) along the side of the test tube. The formation of brown ring at the interface gives positive indication for cardiac glycoside and a violet ring may appear below the brown ring.

*Carbohydrates*

The extract was mixed with Molisch reagent, and then added concentrated H2SO4 along the sides of the test tube to form layers. Appearance of reddish violet ring the interference indicated the presence of carbohydrates. 

*Determination of total carbohydrate, total phenolic and total flavonoid contents*

*Total carbohydrate content*

Total carbohydrate content was quantified using the phenol-sulfuric acid method with glucose as the standard (17). Briefly, appropriately diluted EFA sample and glucose standards were mixed with 500 mL 4% phenol and 2.5 mL of 96% sulfuric acid. Absorbance at 490 nm was measured and carbohydrates in the EFA were expressed as glucose equivalent. The phenol-sulfuric acid method has an accuracy of ±2%.

*Total phenolic content*

Total phenolic contents were evaluated with Folin–Ciocalteu’s phenol reagent (18) using spectrophotometric analysis (Cary 50 Scan UV–Visible apparatus). Briefly, an aliquot (1 mL) of standard solutions of gallic acid at different concentrations or appropriately diluted extracts was added to a 25 mL volumetric flask containing 9 mL of ddH2O. A reagent blank using ddH2O was prepared. One milliliter of Folin and Ciocalteu’s phenol reagent was added to the mixture and shaken. After 5 min, 10 mL of 7% Na2CO3 solution was added with mixing. The solution was then immediately diluted to volume (25 mL) with ddH2O and mixed thoroughly. After incubation for 90 min at 23 °C, the absorbance versus prepared blank was read at 750 nm. Total phenolic contents in medicinal plants were expressed as mg gallic acid equivalents (GAE)/g dry weight. The samples were analyzed in three replications.

*Total flavonoid content*

Total flavonoid contents were measured according to a colorimetric assay (19). A 1 mL aliquot of standard solutions of quercetin at different concentrations or appropriately diluted samples was added to a 10 mL volumetric flask containing 4 mL ddH2O. At zero time, 0.3 mL 5% NaNO2 was added to the flask. After 5 min, 0.3 mL 10% AlCl3 was added. At 6 min, 2 mL of 1 M NaOH was added to the mixture. Immediately, the solution was diluted to volume (10 mL) with ddH2O and mixed thoroughly. Absorbance of the mixture, pink in colour, was determined at 510 nm versus the prepared blank. Total flavonoid contents in medicinal plants were expressed as mg quercetin equivalents (CE)/g dry weight (dw). The samples were analyzed in five replications.

*Animals*

Male Wistar rats (200-250 g; Pasteur institute, Tehran, Iran) were used in this study. Four to six rats were housed in each cage. Tap water and rodent food pellets were available *ad libitum*. The rats were given one week adaptation before experiments at 22 ± 1 °C in a 12 h light/dark cycle. The animals were allowed at least 2 h for adaptation to the new environment (*i.e. *laboratory) before drug administration. Experiments were carried out in a quiet room under dim light between 9:00am and 4:00pm. Seven to twelve rats were used in each treatment group. The animals were used only one time for behavioral testing. Naive rats were used for all behavioral experiments. All procedures were in accordance with the Shahid Beheshti University of Medical Sciences Guidelines for the Care and Use of Laboratory Animals and were approved by the local Research and Medical Ethics Committee.

*Preparation of drugs for administration to animals*

Diazepam hydrochloride was used as the positive control drug (10 mg/2 mL; Daru Pakhsh, Tehran, Iran). It was diluted with normal saline before use. Moreover, different concentrations of EFA were prepared by serial dilution from a stock solution of 70 mg/mL of the extract dissolved in saline. EFA at doses of 2.18-700 mg/kg was used for behavioral assessments. These doses are based on a preliminary study of our ‎group (unpublished data). All drug solutions were prepared freshly on the day of experiment and administered intraperitoneally (IP) in a volume of 1 mL/100 g body weight of rats.

*Behavioral tests*

*The optimum dose of diazepam for behavioral assessments*

‎In order to obtaining optimum dose of diazepam, its different concentrations (0.3-6 mg/kg, IP) were tested in the EPM, open field, and horizontal wire tests (20, 21). 

*Elevated plus-maze test*

Anxiety was assessed using the rat elevated plus-maze test (EPM) (22, 23). The apparatus consists of two open and two closed horizontal perpendicular arms (50 × 10 cm) positioned 40 cm above the floor. The junction of four arms forms a central square platform (10 × 10 cm). Each animal was placed in the junction of open and closed arms facing one of the closed arms and allowed to explore freely for 5 min. The sessions were recorded by a camera positioned right above the maze hanging from the ceiling. Data were obtained using Ethovision software (version 7); a video tracking system for automation of behavioral experiments (Noldus Information Technology, the Netherlands). During the 5 min trial, the behavior of each rat was recorded in terms of ‎the percentage of time spent on ‎open or closed arm [= Total time spent on ‎arm (sec) × 100/300 (sec)] and percentage of entries into open or closed ‎arms (= Total entries into arm × 100/entries to all arms). Increase in the percentage time ‎spent on open arms and/or percentage open ‎arms entries were (was) inferred as the ‎index of lower anxiety behavior. Also, the number of closed arm entries, ‎‎total arm entries, total distance traveled (cm), and velocity were recorded. These ‎parameters reflected animal ‎locomotion and activities (24). The ‎EPM test was assessed in both of repeated and single dose treated groups as follows. ‎

*Effects of repeated treatment with EFA on the EPM*

The repeated administration was performed as following: the rats were randomly ‎divided into 5 groups; the first group was treated with saline (the vehicle), the ‎second group was treated with diazepam (1.2 mg/kg); and the third to fifth ‎groups were treated with the extract of *A. aucheri* at doses of 35, 70 and 175 ‎mg/kg, respectively via intraperitoneal route for 4 days once daily. Each ‎group contained 10-12 rats. ‎Then, the rats were tested ‎in the EPM 24 h after the last dose (Repeated-24 h). ‎ Moreover, in two similar but separate ‎groups, the rats were treated as mentioned above, but the EPM was performed at interval of 48 ‎or 96 h from the last dose (Repeated-48 h, Repeated-96 h, respectively). Totally, fifteen groups were included in the repeated ‎administration study. ‎ After removal of rat, the apparatus was wiped clean with ethanol (10%).

*Effects of single dose treatment with EFA on the EPM* *‎*

In single dose treatment, a separate group of rats ‎ received a single ‎IP ‎injection of saline, diazepam or EFA ‎at doses of 35, 70, and 175 mg/kg; one ‎hour before the EPM test (Single-1 h). ‎ Each group contained 12 rats.‎ After removal of rat, the apparatus was wiped clean with ethanol (10%).

*Open field test*

The acute sedative activity was investigated by recording spontaneous locomotor activity of rat in an open field apparatus (25). Spontaneous locomotor activity was determined in individual rat which was placed in the center of an open field apparatus (45 × 45 cm), by a camera positioned right above the apparatus hanging from the ceiling. The data were obtained using Ethovision software (version 7) (see above). Locomotor activity was defined as total distance traveled (cm) by rat during a 30 min period immediately after acute IP injection of saline, diazepam (3 mg/kg), or EFA (2.18, 8.75, 17.5, 35, 70, 175, 350 and 700 mg/kg) in groups of 7 rats. In this test, sedation was defined by a significant decrease in locomotor activity. After each trial, the open field apparatus was wiped clean with ethanol (10%).

*Horizontal wire test*

Immediately after the open field test, the rats in each group including single dose of EFA (at 17.5, 35, 70, 175, 350, and 700 mg/kg; n = 7), diazepam (6 mg/kg; n = 7) or saline (n = 7) were subjected to a horizontal wire test, as described by Hui *et al.*, with minor modifications (26). Briefly, the rats were lifted by the tail and allowed to grasp a horizontally strung wire (2 mm diameter, 40 cm long, placed 60 cm above a table) with their forepaws. When the rat grasped the wire with both forepaws, its tail was gently released. The number of rats from each treatment group that did not grasp the wire with their forepaws or actively grasped the wire with at least one hind paw within 3 sec was recorded; myorelaxant agents are known to impair the ability of rat to grasp the wire. After each trial, the horizontal wire apparatus was wiped clean with ethanol (10%).

*Statistical analysis*

All of the behavioral tests plus-maze, open field and horizontal wire test scores were subjected to one way ANOVA with Newman-keuls *t* post-hoc tests for differences between individual groups, using GraphPad Prism software (version 7, Graphpad Software Inc., USA). The extract dose, that produced the half maximal response (=ED50) in the open field test was calculated using linear regression. A *p*-value of less than 0.05 (*p* < 0.05) was considered significant. 

## Results

*Phytochemical screening*

Phytochemical analysis of the aqueous extract revealed the presence of phenolic compounds, ‎flavonoids, and polysaccharides as well as the absence of alkaloids, tannins, saponins, sterols, and ‎cardiac glycosides.‎

*Total carbohydrate, total phenolic and total flavonoid contents*

Total content of polysaccharides, flavonoids, and phenolic compounds were quantified in the aqueous extract; the results are shown in Table 1.

*Behavioral tests*

*The optimum dose of diazepam for behavioral assessments*

Effects of diazepam on the EPM, open field, and horizontal wire tests were presented in Figure 1. ANOVA showed that diazepam at dose of 1.2 mg/kg significantly increased percentage of time spent on open arms (*p *< 0.05), at dose of 3 mg/kg decreased locomotor activity (*p *< 0.01), and at dose of 6 mg/kg has myorelaxant effect in rats (*p *< 0.05). So, we used these doses of diazepam as positive control drug.

*Effects of repeated treatment with EFA on anxiety*

One-way ANOVA indicated that there were statistically significant differences between treatment groups in the percentage of time spent on open arms [*F*(4,53) = 4.06; *p* = 0.01] and of open arms entries [*F*(4,53) = 5.10; *p* = 0.004], in the Repeated-24 h (Figure 2). The Newman-keuls *t* post-hoc analysis revealed that the EFA at dose of 35 mg/kg significantly increased percentage of time spent on open arms and of open arms entries, compared with saline control. Furthermore, there were significant differences in the percentage of time spent on closed arms [*F*(4,53) = 4.75; *p* = 0.006] and of closed arms entries [*F*(4,53) = 4.43; *p* = 0.007], in Repeated-24 h. The Newman-keuls *t* post-hoc analysis revealed that EFA at the same dose (35 mg/kg) significantly decreased percentage of time spent on closed arms and of closed arms entries (45.52 ± 4.16 for EFA versus 66.24 ± 4.89 for saline, *p* < 0.05; 23.50 ± 2.66 for EFA versus 36.11 ± 1.08 for saline, *p* < 0.05, respectively).

However, the increases in the open arm activity by EFA at higher doses (70 and 175 mg/kg) didn´t attain conventional levels of significance ‎ (Figure 2). In addition, there was a significant effect in open arm activity of rats ‎ in Repeated-48 h [*F*(4,53) = 2.63; *p *= 0.04]. The Newman-keuls *t* post-hoc analysis showed that EFA at 175 mg/kg increased the percentage of time spent on the open arms, compared with saline-treated group ‎ (Figure 2). Finally, one-way ANOVA revealed that in the repeated treatment groups which were tested 96 h after the last dose, there was no significant difference in open arm activity between EFA and saline groups 

(Figure 2). Unlike its initial effect in acute dose (See Figure 1), however, diazepam couldn´t change open arm activity after repeated treatment.

*Effects of single dose treatment with EFA on anxiety*

There was a significant difference in the percentage of time spent on open arms (*F*(4,55) = 4.56; *p* = 0.001) (Figure 3). Newman-keuls *t *post-hoc comparison revealed that EFA (at 175 mg/kg) or diazepam increased percentage of time spent on open arms, compared with control. There was no significant difference with respect to the percentage of open arms entries.

*Effects of repeated treatment with EFA on locomotor activity in EPM test*

There was a significant effect of treatment on the total distance traveled by rats in the elevated plus maze (*F* (4,53) = 5.80, *p* < 0.002), in Repeated-24 h (Table 2). Newman-keuls *t *post-hoc tests revealed a significant difference in distance between EFA at 175 mg/kg or diazepam, when compared to control group. A similar difference was also observed in reference to velocity in the maze (*F*(4,53) = 4.75, *p* = 0.005) (Table 2). Newman-keuls *t* post-hoc tests revealed a significant difference in velocity between EFA at 175 mg/kg and saline control group.

*Effects of single dose treatment with EFA on locomotor activity in open field test*

There was a significant difference between groups in total distance traveled by rats in the open field [*F*(9,60) = 5.93; *p *< 0.0001] (Figure 4). Newman-keuls *t* post-hoc showed a significant difference in distance traveled between EFA at doses equal or greater than 17.5 mg/kg, or diazepam; and control group. Linear regression analysis confirmed a dose-dependent effect of EFA on locomotor activity *i.e.* increasing the dose of EFA caused a reduction in locomotor activity. Linear regression revealed a 50% reduction in locomotor activity of EFA at 194 mg/kg (=ED50) compared with control group (data not shown) [*F*(9,60) *= *83.95*; p < *0.0001].

*Effects of EFA on horizontal wire test*

ANOVA indicated that there was a significant effect of treatment on the percentage of rats grasping the wire [*F*(7.53) = 4.98; *p* = 0.0002] (Figure 5). Newman-keuls *t* post-hoc tests revealed a significant difference in percent of rats grasping the wire between diazepam 

(6 mg/kg) and control group. Meanwhile, there was no significant difference among EFA and saline groups. ‎ 

## Discussion

The present study demonstrated that the aqueous extract of *A. aucheri* flowers had anxiolytic and sedative activities in rat. These activities revealed by increased open arm exploratory behavior and decreased locomotor activity of animals, respectively. To our knowledge, such behavioral effects have not been reported previously for *A. aucheri* or other plants of the *Alcea L*. genus. For evaluation of effects of the extract on anxiety, we used EPM. The EPM is one of the most widely indicated models for study of animal anxiety. This test uses natural stimuli, *i.e.* the fear of a new, brightly-lit open space, and the fear of balancing on a relatively narrow raised platform (27, 28). This test has been validated for study of anxiety in both of rats and mice (22, 29). Therefore, we used this test to assess anxiolytic potential of EFA in rats. In EPM test, an increase in open arm activity (duration and/or entries) reflects anti-anxiety behavior (23). Standard anxiolytic agents such as benzodiazepines, via activating GABA_A_ receptor-complex, increase percentage of time spent on open arms and/or percentage of open arms entries. Therefore, in this study, diazepam was used as a positive control drug. 

The findings of the present study demonstrated that treatment with the aqueous extract was effective in inducing anxiolytic effects with single acute treatment, a phenomenon that remained after repeated treatment. It is well known that when single dose of a substance induces a pharmacologic effect, its repeated doses with a longer period, at the same dose and through the same route; usually will increase the effect (30). Besides, although the repeated treatment with extract has anxiolytic effect 24 h after the last dose (Repeated-24 h); we noticed that the trend of the anxiolytic effect of EFA is clearly different from that in acute treatment (See Figure 2, panel A; Figure 3). In the first group, increasing the dose didn´t increase its effect on open arm. Therefore, unlike its acute single dose, repeated higher doses of the EFA didn´t produce anxiolytic effect in elevated plus-maze. The cause (s) of such differences is not clear. The authors believed that possibly various factors acting separately or in combination may be responsible. Some probable factors include change in the pharmacokinetic profile of active ingredient (s) following repeated administration of drug, desensitization to drug effects and, finally decreased locomotor activity in the EPM test. The last one is based on the results presented in Table 2, which revealed that repeated dosing of EFA significantly reduced total distance traveled and velocity, and so locomotor activity in the EPM test (31). Consequently, the decreased locomotor activity in turn, would interfere with the performance of rats in anxiety test. Based on this assumption, though one can describe the loss of initial anxiolytic response to extract (at 175 mg/kg); however, this couldn’t explain the difference in the trend between two groups (Single-1 h and Repeated-24 h). Therefore, other causes would be considered, as well. Another possible factor may be the phenomenon of "desensitization", that is attenuation of responses to drug. After reaching an initial high level, the response diminishes over seconds or minutes, even in the continued presence of the agonist (30). This usually occurs when high or repeated doses were used to produce a pharmacological effect. This phenomenon may be supported by re-appearance of initial anxiolytic effect of this dose, *i.e.* increase in open arm activity, after 48 h of termination of its administration (Repeated-48 h) (Figures 2 and 3). 

In addition, unlike the *Alcea* extract, diazepam as the control positive drug, failed ‎to induce anxiolytic activity after repeated treatment (Figure 2). Possibly, this ‎indicates that under the conditions of the present study, duration of the ‎anxiolytic effect of EFA is longer than that of diazepam; and difference in ‎kinetic and/or dynamic profile (s) may be contributed.‎

Evaluation of locomotor activity in open field showed that acute EFA treatment at doses of ≥ 17.5 induced a dose-dependent reduction in locomotor activity of rats. In the other word, EFA produced a sedative effect, acutely. However, assessment of locomotor measures in the EPM test in Single-1 h (24, 31), didn´t reveal any reduction in locomotor activity of animals (Table 2). According to these results and different testing times of two tests, it could be concluded that the acute sedative effect of EFA was abolished in a time between the time of open field test and EPM test (See Methods). Based on the later finding, it could be understood that locomotor effect of the extract could not ‎confound interpretation of behavioral changes that are used as indices of ‎anxiety reduction in EPM in Single-1 h group. Meanwhile, with repeated dosing, ‎EFA decreased velocity and the distance traveled by ‎rats, *i.e.* decreased locomotor activity; in Repeated-24 h (Table 2)**. **

‎In horizontal wire test we found that unlike diazepam, *A. aucheri* did not show myorelaxant effect (Figure 5). If this finding from animal studies has direct relevance to therapeutic application of *A. auheri*, this may provide a pharmacological advantage of *A. aucheri* over the benzodiazepine anxiolytic drugs.

Collectively, the findings of the present study indicated that the aqueous extract of *A. aucheri *‎flowers produced CNS depressant effects in rats. With the experimental tests used in this work, it is not possible to elucidate the action mechanism through which EFA exerts both effects. Therefore, it is necessary to develop biochemical and pharmacological studies that allow us to establish if the effects here reported are a consequence of the separate activation of nervous structures by one chemical compound by itself, or if the biological activities are produced by different secondary metabolites of the plant. 

Phytochemical screening confirmed the presence of phenolics, ‎flavonoids, and polysaccharides in the aqueous ‎extract from the flowers of *A. aucheri* used in this work. ‎Quantitative analysis, revealed that phenolic compounds constitute the major component identified. This group of secondary metabolites constitutes about 13.6% ‎of dry weight of the extract (Table 1). Examples of common groups of plant phenolics include cinnamic acid derivatives (phenolic acids) and flavonoid compounds (32). ‎ The flavonoids identified in ‎the plants of the genus *Alcea (Althea)* are mainly derivatives of kaempferol, quercetin, luteolin, and myricetin (13, 33). In the previously published studies, it had been confirmed that flavonoids such as quercetin and kaempferol are responsible for anxiolytic effects of some plants (34-36). So, the flavonoids may have contributed to ‎the observed central depressant effects of EFA. This effect has been attributed to the affinity of flavonoids for the central benzodiazepine receptors (37-39). Furthermore, a sedative effect on central nervous system has been shown for quercetin (40). However, there are different reports on effects of the flavonoid kaempferol on locomotor activity in animal models (34, 41).‎

Beside flavonoids, the other main classes of phenolic compounds present in the plants of *Alcea* genus ‎are the ‎phenolic acids. The phenolic acids which have been previously identified ‎in flowers of *A. rosea* include salicylic,‎ vanillic, ferulic, syringic, caffeic, p-hydroxybenzoic, p-coumaric, and p-‎hydroxyphenylacetic acids (33). Pharmacological studies have demonstrated that ‎phenolic acids display central nervous system depressor activities (42, 43)‎‏. Therefore, the phenolic acids present in the EFA may be accounted for the observed effects, as well. By using an *in-vitro* ‎screening approach to identify naturally occurring phytochemical GABA agonists, Scheepens *et al.* showed that the plant secondary metabolite p-‎coumaric acid to have significant GABAergic activity, and the effect was ‎blocked by co-administration of the specific GABA-receptor antagonist, ‎picrotoxin. Moreover, oral administration of p-coumaric acid to rodents ‎induced a significant ‎anxiolytic effect *in-vivo* as measured using the elevated ‎plus paradigm ‎‎(44).‎ Furthermore, some studies demonstrated that caffeic acid ‎ induced anxiolytic effects in rodents, *i.e.* increased the number of ‎entries and the time spent in the open arms on plus maze ‎‎(45, 46).‎

In addition, other compounds which also may have contributed to the ‎observed effects are the polysaccharides present in our extract. Pharmacological studies have demonstrated that polysaccharides from the plants display central depressant activity (47-49). It has been reported previously that polysaccharides isolated from a methanolic extract of *Ipomoea tyrianthina L.* roots exhibited potentiation of hypnosis induced by pentobarbital and released GABA from mouse cortical brain slices in mice. They indicated that polysaccharides of *I. trianthina* possess anxiolytic effects and could have potential sedative effect, probably through a GABAergic system (48). Such effects were also observed with an ethyl acetate extract from the roots of *I. stans* (49). 

However, many authors believe that the certain pharmacological activity of the plants is, in fact, the consequence of the present combination of chemical compounds in a plant. Therefore, possibly, the anxiolytic and sedative activities observed in this work were not only dependent on the flavonoid, phenolic acids, and polysaccharide content, and the other compounds belonging to different phytochemical classes were involved in the biological response observed in this work. ‎ Accordingly, the observed central depressant effects of *A. aucheri* is probably the consequence of the blend of all the compounds present in it.‎ Nevertheless, possible active ingredient (s) responsible for the anxiolytic and/or sedative effects of *A. aucheri* need to be isolated and identified in forthcoming studies. 

In conclusion, our results showed that the *A. aucheri* flowers have anxiolytic-like and sedative effects in rats, with no myorelaxant effect. However, the exact mechanism (s) related to the active ingredient (s) in *A. aucheri* extract should be elucidated in future studies. Additional investigation by our group is in progress.
